# Analysis of the Prevalence of Bacterial Pathogens and Antimicrobial Resistance Patterns of *Edwardsiella piscicida* in Largemouth Bass (*Micropterus salmoides*) from Guangdong, China

**DOI:** 10.3390/pathogens13110987

**Published:** 2024-11-12

**Authors:** Weimin Huang, Changyi Lin, Caiyi Wen, Biao Jiang, Youlu Su

**Affiliations:** Guangzhou Key Laboratory of Aquatic Animal Diseases and Waterfowl Breeding, Innovative Institute of Animal Healthy Breeding, College of Animal Sciences and Technology, Zhongkai University of Agriculture and Engineering, Guangzhou 510222, China; huangweimin@zhku.edu.cn (W.H.); linchangyi@zhku.edu.cn (C.L.); wencaiyi@zhku.edu.cn (C.W.)

**Keywords:** largemouth bass, epidemiological investigation, *Edwardsiella piscicida*, drug sensitivity analysis

## Abstract

To gain insights into the prevalence and antimicrobial resistance patterns of major bacterial pathogens affecting largemouth bass (*Micropterus salmoides*) in the Pearl River Delta (PRD) region, Guangdong, China, a study was conducted from August 2021 to July 2022. During this period, bacteria were isolated and identified from the internal organs of diseased largemouth bass within the PRD region. The antimicrobial resistance patterns of 11 antibiotics approved for use in aquaculture in China were analyzed in 80 strains of *Edwardsiella piscicida* using the microbroth dilution method. The results showed that 151 bacterial isolates were obtained from 532 samples, with *E. piscicida* (17.29%, 92/532), *Aeromonas veronii* (4.70%, 25/532), and *Nocardia seriolae* (2.26%, 12/532) being the main pathogens. Notably, *E. piscicida* accounted for the highest proportion of all isolated bacteria, reaching 60.92% (92/151), and mainly occurred from November to April, accounting for 68.48% (63/92) of the cases. The symptoms in largemouth bass infected with *E. piscicida* included ascites, enteritis, and hemorrhaging of tissues and organs. The drug sensitivity results showed that the resistance rates of all *E. piscicida* strains to ciprofloxacin, all sulfonamides, thiamphenicol, florfenicol, enrofloxacin, doxycycline, flumequine, and neomycin were 96.25%, 60–63%, 56.25%, 43.75%, 40%, 32.5%, 16.25%, and 1.25%, respectively. In addition, 76.25% (61/80) of these strains demonstrated resistance to more than two types of antibiotics. Cluster analysis revealed 23 antibiotic types (A–W) among the 80 isolates, which were clustered into two groups. Therefore, tailored antibiotic treatment based on regional antimicrobial resistance patterns is essential for effective disease management. The findings indicate that in the event of an *Edwardsiella* infection in largemouth bass, neomycin, doxycycline, and flumequine are viable treatment options. Alternatively, one may choose drugs that are effective as determined by clinical drug sensitivity testing.

## 1. Introduction

The largemouth bass (*Micropterus salmoides*), also known as the California bass, is a member of the Centrarchidae family and native to the Mississippi River Basin in the United States [1]. The largemouth bass has held an important position in China’s aquaculture industry due to its delicious meat and lack of intramuscular bones [2]. In 2023, the production of freshwater bass (mainly, largemouth bass) in China exceeded 880,000 tons, with Guangdong accounting for approximately 41.4% of the total production [3]. In recent years, despite significant progress in breeding technology, the development of the largemouth bass farming industry has encountered numerous issues. The increasingly severe disease issues have become the main bottleneck hindering the development of the largemouth bass farming industry [4]. Among them, the prevalence of bacterial diseases is particularly prominent, and their severity has prompted extensive antibiotic use by farmers, leading to prominent issues linked to drug residues [5]. Common pathogenic bacteria affecting largemouth bass include *Edwardsiella*, *Aeromonas*, *Nocardia*, among others [6]. In our preliminary research, we found that the largemouth bass aquaculture industry in the Pearl River Delta (PRD) region was also deeply affected by bacterial diseases, with *Edwardsiella piscicida* infection being the most serious.

The genus *Edwardsiella* was established in 1965, when *Edwardsiella tarda* was first isolated from human feces and identified as a member of the Enterobacteriaceae family [7]. In 1993, Francis-Floyd et al. [8] isolated *E. tarda* from largemouth bass in Florida, U.S.A., and since then, it has been found in various parts of the world. However, in 2012, a new species that had previously been misidentified as *E. tarda* was renamed *Edwardsiella piscicida*, based on its phenotypic and genetic characteristics [9]. *E. piscicida* is non-host-specific and has the ability to infect a wide range of marine and freshwater fish species. In recent years, *E. piscicida* has been isolated from various fish species, including seabass (*Lateolabrax maculatus*) [10], tilapia (*Oreochromis mossambicus*) [11], *Siniperca chuatsi* [12], and crucian carp (*Carassius auratus*) [13], and has been identified as the causative agent of *Edwardsiella* septicemia, also known as Edwardsiellosis. The affected fish exhibit symptoms such as spiral swimming at the surface, ulceration of the body surface, abdominal swelling, and redness of the anus. Dissection of the abdominal cavity reveals pathological changes, including large amounts of ascites and enlarged viscera [14]. Edwardsiosis can occur throughout the year without any apparent seasonality; it can occur at water temperatures of 15 °C, with a higher incidence of infection in the water temperature range of 25–30 °C, which typically makes it more prevalent during spring and summer [15]. Currently, research on this disease is primarily focused on case reports and pathogenicity studies. Nonetheless, in the PRD region, a major area for the largemouth bass, there is a lack of epidemiological investigations on bacterial diseases, particularly Edwardsiellosis. Understanding the epidemiological patterns of bacterial diseases in largemouth bass is fundamental to establishing prevention and control strategies.

At present, there are no commercially available vaccines for Edwardsiellosis in largemouth bass, which makes antimicrobial drugs the primary method for preventing and treating this disease. The extent of losses due to Edwardsiellosis during farming negatively correlates with the duration of intervention and treatment. However, because *E. piscicida* is an intracellular bacterium, most antibiotics are ineffective against it. Currently, the majority of treatments for Edwardsiellosis in largemouth bass involve the external disinfection of nets with chlorine dioxide, coupled with the continuous feeding of enrofloxacin powder mixtures for seven days, which can significantly reduce the mortality rates [16]. However, it should be noted that the overuse of antibiotics has resulted in the emergence of highly resistant bacterial species, which is also the main reason for the difficulty in disease prevention and control [17]. Kashif Manzoor found that antibiotic misuse leads to the emergence of the ABR gene in *Edwardsiella*, which can increase drug resistance [18]. Recent studies have identified 87 strains of *E. piscicida* that are resistant to multiple antibiotics [19]. Therefore, understanding the antimicrobial resistance patterns of bacteria in the region is crucial for offering a reliable reference for the precise administration of medications.

The primary aim of this study was to identify the bacterial diseases that predominantly affect largemouth bass in the PRD region. Furthermore, we intended to undertake a thorough examination of the epidemiological situation associated with Edwardsiellosis. Additionally, we aimed to conduct a comprehensive assessment of the antimicrobial resistance of *E. piscicida*. We anticipate that the findings from this study will provide invaluable insights for the effective prevention and control of *E. piscicida* infections in farmed largemouth bass.

## 2. Materials and Methods

### 2.1. Sample Collection

In this study, a total of 532 diseased largemouth bass were randomly collected from various largemouth bass farms in the PRD region between August 2021 and July 2022. The locations of the sampling sites are summarized in Table 1. Prior to the inclusion of fish in this study, the farm owners were asked if they were willing to participate in the research activities, and their verbal consent was obtained. All protocols involving fish handling during the experiments were approved by the Experimental Animal Ethics Committee of Zhongkai University of Agricultural Engineering (Approval Code: ZK20210402).

### 2.2. Isolation and Identification

After anesthesia with 100 mg/L of eugenol solution (Changshu Shangchi Dental Material, Suzhou, China), the largemouth bass were sterilized using 75% alcohol and dissected using sterile equipment to expose the abdominal cavity. Bacteria were isolated from the liver, spleen, and kidneys using an inoculation loop, streaked on brain heart infusion (BHI, Huankai Microbial Technology, Guangzhou, China) agar plates, and incubated at 30 °C for 24–72 h in a thermostatic biochemical incubator. Subsequently, a single bacterial colony was purified on a new BHI agar plate, and then the bacteria were preserved in BHI medium containing 25% sterile glycerol at −80 °C in an ultra-low-temperature freezer.

We used universal primers (upstream primer 16S-27F: 5′AGAGTTTGATCCTGGCTCAG-3′; downstream primer 16S-1492R: 5’-GGTTACCTTGTTACGACTT-3’) for bacterial *16S rRNA* in the following polymerase chain reaction (PCR). The PCR amplification system and protocol were as follows. In a 20 μL reaction mixture, 10 μL of 2X M5 HiPer plus Taq HiFi PCR mix (Mei5 Biotechnology, Beijing, China) was used, with 0.5 μL of the primers F and R. The amplification program consisted in an incubation at 95 °C for 3 min (initial denaturation); 36 cycles at 94 °C for 25 s (denaturation), 55 °C for 25 s (annealing), and 72 °C for 10 s (extension); and an incubation at 72 °C for 5 min (final extension). The amplification products were then detected by 1% agarose gel electrophoresis and sent to Guangzhou Tianyi Huiyuan Biotechnology Co., Ltd. (Guangzhou, China) for sequencing services. After sequencing, the obtained 16S rDNA sequences were compared with bacterial gene sequences stored in the NCBI database using the Basic Local Alignment Search Tool (https://blast.ncbi.nlm.nih.gov/Blast.cgi (accessed on 13 August 2022)). For the *Edwardsiella* sp., we conducted another round of amplification, sequencing, and comparison using *gyrB* (NZ_CP090968.1, upstream primer *gyrB*-F: 5′-GAAGTCATCATGACCGTTCTGCA-3′; downstream primer *gyrB*-R: 5’-AGCAGGGTACGGATGTGCGAGCC-3′).

### 2.3. Antimicrobial Sensitivity Assay

According to the approval of the Ministry of Agriculture and Rural Development of the People’s Republic of China, 11 antibiotics (thiamphenicol, florfenicol, flumequine, enrofloxacin, ciprofloxacin, doxycycline, neomycin, sodium sulfamonomethoxine, sulfamethoxazole, sulfadiazine, and sulfamethazine; Macklin Biochemical Technology, Shanghai, China) approved for use in aquaculture were chosen to carry out the antimicrobial sensitivity assay. In total, 80 *E. piscicida* isolates were tested for resistance to these 11 antibiotics using the microbroth method, with *Escherichia coli* (ATCC 25922) selected as the quality control strain. The 11 antibiotics were dissolved to an initial concentration of 2048 μg/mL and then diluted to the experimental concentrations. The *E. piscicida* isolates were cultured in Mueller–Hinton (MH) broth (Huankai Microbial Technology, Guangzhou, China) at 30 °C for 16 h. Subsequently, the concentration of the bacterial suspensions was adjusted using MH broth to a turbidimetric level of 0.5 McFarland (MCF).

The drug sensitization test was conducted using 100 μL of bacterial solution and 100 μL of the drug solutions at different concentrations, which were added to the wells of a sterile 96-well plate. The final concentration of the drugs in each well is shown in Table 2. Two wells were used as a positive control (bacterial solution + broth), and two others as a negative control (sterile water + broth). The plates were sealed with a sterile breathable film and incubated in a biochemical incubator at a constant temperature of 30 °C for 18–22 h. The results were recorded to determine whether the isolates were resistant to the 11 antibiotics in accordance with the antibiotic susceptibility testing standards of the Clinical and Laboratory Standards Institute (CLSI M100 Ed34). The bacteria were categorized as resistant, moderately susceptible, or susceptible.

### 2.4. Antibiotic Resistance Rates and Antibiogram Analysis

The resistance rates to the 11 antibiotics were analyzed using WHONET microbiology laboratory database software and IBM SPSS Statistics for Windows (version 27.0; IBM Corporation, Armonk, NY, USA). The resistance of all isolates to each of the 11 tested antibiotics was recorded.

### 2.5. Data Analysis

Statistical analyses were performed using IBM SPSS Statistics for Windows (version 27.0). The gradient difference between the MIC and the intermediate inhibitory concentration in the antimicrobial sensitivity assay was used as the variable in a clustering analysis of antibiogram types performed using Ward’s clustering method.

## 3. Results

### 3.1. Identification of E. piscicida Isolated from Largemouth Bass

**Figure 1:**
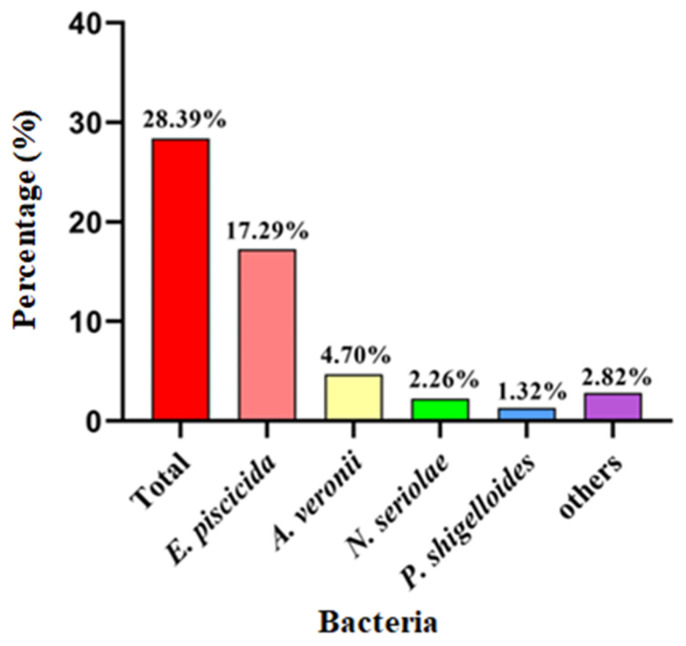
The percentage of pathogenic bacteria isolated from the examined diseased largemouth bass.

In total, 151 bacterial isolates were collected from the 532 examined largemouth bass from the PRD region. As shown in Figure 1, the pathogenic bacteria isolated mainly included *E. piscicida* (17.29%, 92/532), *Aeromonas veronii* (4.70%, 25/532), *Nocardia seriolae* (2.26%, 12/532), and *Plesiomonas shigelloides* (1.32%, 7/532).

**Figure 2:**
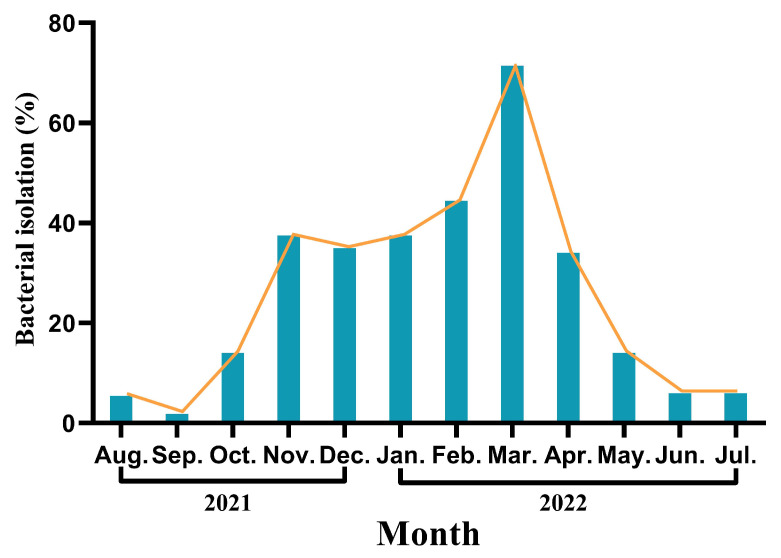
The monthly analysis results of *Edwardsiella piscicida*.

*E. piscicida* was the most commonly isolated organism from August 2021 to July 2022 and mainly occurred from November 2021 to April 2022, accounting for 68.48% of the infections in that period (63/92). As shown in Figure 2, the prevalence of *E. piscicida* tended to decrease from August to September 2021, especially in September, which had the lowest isolation rate of the entire study period, corresponding to only 1.85% (1/54). However, from September until March 2022, the isolation rate consistently increased. Notably, during the four-month period from November 2021 to February 2022, the isolation rate of *E. piscicida* was steady at approximately 40%. Subsequently, in March 2022, the isolation rate sharply increased to 71.43% (15/21) but then decreased again each month.

### 3.2. Pathological Symptoms in Largemouth Bass Infected with E. piscicida

**Figure 3:**
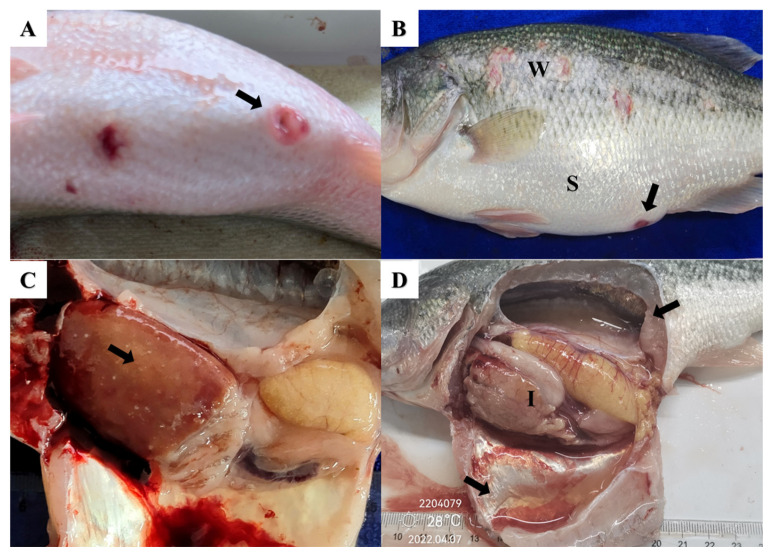
Clinical signs in largemouth bass infected with *Edwardsiella piscicida*. (**A**) Anal red swelling (arrow); (**B**) abdominal swelling (S), wounds were accompanied by the shedding of fish scales (W) and anal red swelling (arrow); (**C**) white nodules in the liver (arrow); (**D**) effusion in the abdominal cavity and swim bladder cavity (arrows) and liver whitening and ischemia (I).

The clinical symptoms in largemouth bass naturally infected with *E. piscicida* include obvious abdominal swelling, anal redness, and wounds on the body surface characterized by whiplash-like shedding of the scales (Figure 3A,B). Further dissection and observation revealed fluid accumulation in the abdominal cavity and swim bladder, in addition to enlargement of several internal organs that presented distinct white nodules (Figure 3C,D).

### 3.3. Drug Sensitivity Test Results for E. piscicida

**Figure 4:**
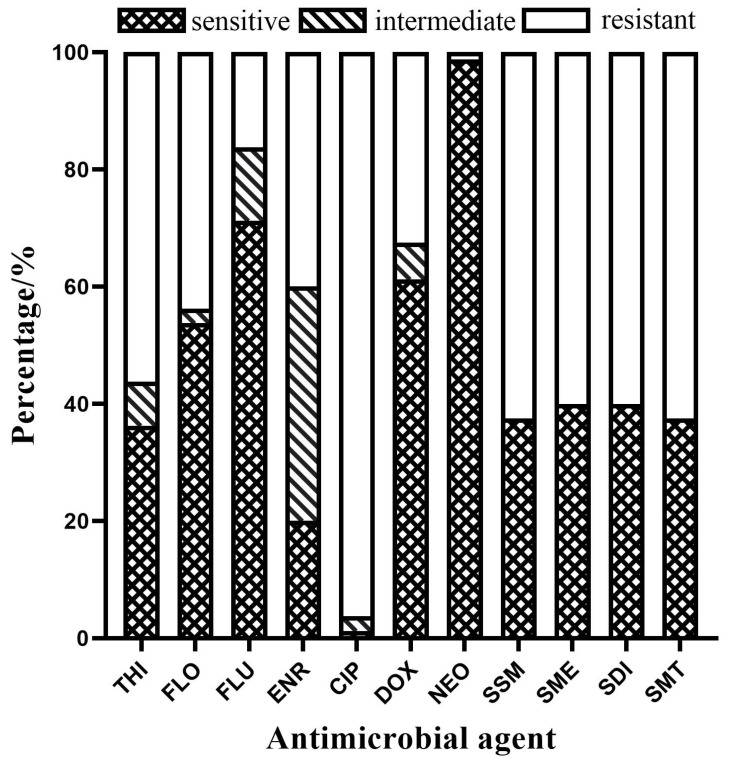
The resistance rates of *Edwardsiella piscicida* to 11 antimicrobial agents. Abbreviations: THI, thiamphenicol; FLO, florfenicol; FLU, flumequine; ENR, enrofloxacin; CIP, ciprofloxacin; DOX, doxycycline; NEO, neomycin; SSM, sodium sulfamonomethoxine; SME, sulfamethoxazole; SDI, sulfadiazine; SMT, sulfamethazine.

There were differences in sensitivity to the 11 antimicrobial agents among the 80 *E. piscicida* isolates. As shown in Figure 4, the resistance rates of all 80 *E. piscicida* isolates to ciprofloxacin, all sulfonamides, thiamphenicol, florfenicol, enrofloxacin, doxycycline, flumequine, and neomycin were 96.25%, 60–63%, 56.25%, 43.75%, 40.00%, 32.50%, 16.25%, and 1.25%, respectively. None of the isolates was sensitive to all the antibiotics.

### 3.4. Antibiogram Types of the E. piscicida Strains

Overall, the 80 isolates of *E. piscicida* corresponded to 23 antibiogram types (A–W) (Table 3), and 28.75% (23/80) of the strains exhibited antibiotic resistance. Among them, type D was the largest group with 19 isolates that were resistant only to ciprofloxacin, whereas types E, H, I, J, and Q each contained ≥5 isolates, types A, B, G, and L contained 2, 3, 3, and 2 isolates, respectively, and types C, F, K, M, N, O, P, R, S, T, U, V, and W each contained only 1 isolate. All resistance profiles, with the exception of M, were susceptible to neomycin.

### 3.5. Cluster Analysis of the Susceptibility of the E. piscicida Isolates to Different Antibiotics

**Figure 5:**
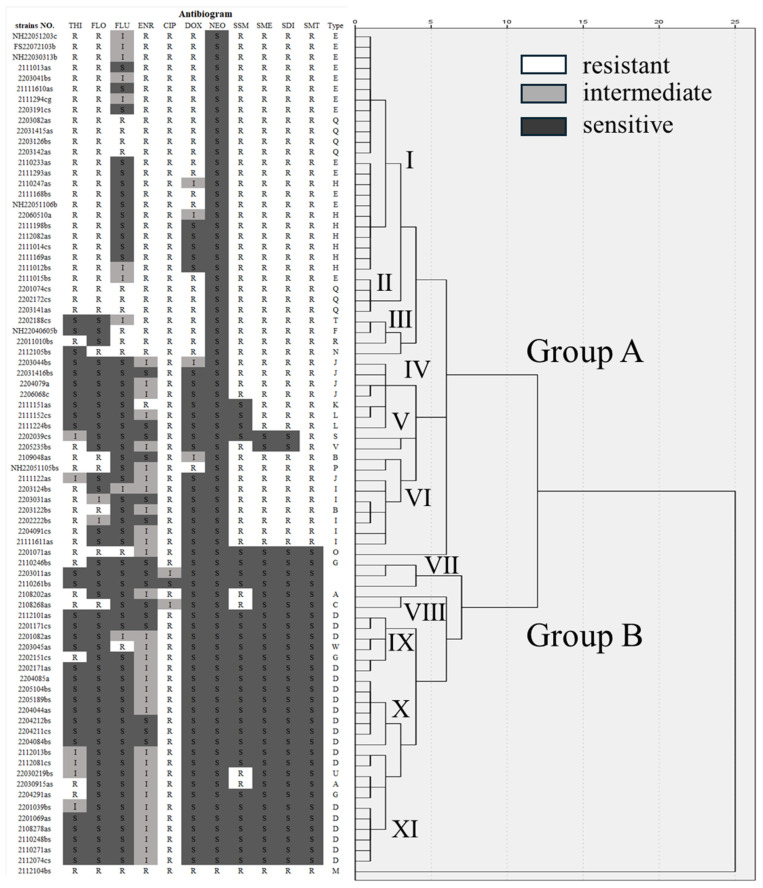
Antibiogram and cluster analysis of the 80 *Edwardsiella piscicida* strains. Abbreviations: THI, thiamphenicol; FLO, florfenicol; FLU, flumequine; ENR, enrofloxacin; CIP, ciprofloxacin; DOX, doxycycline; NEO, neomycin; SSM, sodium sulfamonomethoxine; SME, sulfamethoxazole; SDI, sulfadiazine; SMT, sulfamethazine.

The gradient difference between the MIC and the intermediate inhibitory concentration in the antimicrobial sensitivity assay was used as the variable in a clustering analysis, and two groups were identified considering the antibiograms of the 80 *E. piscicida* strains (Group A and B), which could further be subdivided into 11 subgroups (I–XI) (Figure 5). When the square of the Euclidean distance was 3, the isolates formed 11 subgroups (I–XI). When the square of the Euclidean distance was 6, subgroups I to VI, along with strain 2201071as, clustered as a bundle into Group A, at a distance of 7, and subgroups VII to XI clustered into Group B. The two large groups A and B contained 16 and 6 drug-resistant spectral phenotypes, respectively, with spectral phenotype rates of 69.57% and 23.09%. The subgroups I–XI contained three, two, four, three, two, four, one, two, three, one, and four resistance phenotypes, respectively. In addition, the strain 2112104bs did not belong to any group, whereas two isolates (2203011as, 2110261bs) in Group B XI were susceptible to all antibiotics.

Differences in drug resistance between the two groups of *E. piscicida* were mainly reflected in resistance to sulfonamides. Overall, the isolates with high resistance to these four antimicrobials were mainly concentrated in Group A, while the isolates in Group B had low resistance to the other 10 antibiotics, except for high resistance to ciprofloxacin. Further subdivision of Group A revealed that the subgroups I, II, and III had higher resistance rates to methicillin, florfenicol, enrofloxacin, ciprofloxacin, and doxycycline than the other subgroups.

## 4. Discussion

In view of the frequent occurrence of bacterial diseases in largemouth bass and the insufficient epidemiological data, our team conducted an epidemiological investigation of bacterial diseases in the PRD region from August 2021 to July 2022. In the present study, 532 diseased largemouth bass were collected, and 151 bacterial isolates were identified. The main pathogenic bacteria in largemouth bass were *E. piscicida*, *A. veronii*, and *N. seriolae*, which is in agreement with a previous report by Chen [20]. In recent years, numerous reports indicated that the prevalence and spread of *A. veronii* have surpassed those of *Aeromonas hydrophila*, which was considered the main causative agent of aeromoniasis in China [20,21]. Deng et al. also found that *A. veronii* was the most common bacterium isolated under normal culture conditions whether from fish or from the environment [22]. The majority of *A. veronii* strains isolated from largemouth bass in this study further confirm that *A. veronii* is the primary pathogen responsible for aeromoniasis in these fish. In the PRD region, the infection rate of nocardiosis is only 2.26%, lower than that reported in some previous surveys [23,24]. This may be due to the fact that largemouth bass have almost entirely transitioned from being fed frozen fresh fish to being fed commercial feed in recent years. However, because *N. seriolae* is an intracellular bacterium with a long incubation period, its infection is considered the most difficult bacterial disease to prevent and control. We must be vigilant of the potential harm that nocardiosis can inflict.

In this study, *E. piscicida* was the pathogen with the highest isolation rate from largemouth bass in the PRD region. The clinical signs of *E. piscicida* infection in largemouth bass included fluid accumulation in the common abdominal cavity and swim bladder, whitish ischemia of the liver, and enlargement of the spleen and kidneys, accompanied by enteritis, which is similar to previously reported findings regarding this disease [25,26]. In terms of temporal distribution, the isolation rate of *E. piscicida* exhibited distinct seasonal variations. However, the highest incidence of *E. piscicida* infections in fish occurred during the months with relatively lower water temperatures (from November to April). This result differs from that of previous reports by Wyatt and Esteve et al., which documented bacterial outbreaks from May to November [27,28] but not from December to early April. This discrepancy may be related to the higher temperatures in the PRD region, as some virulence factors of *E. piscicida* are activated at temperatures ranging from 23 to 35 °C [29,30]. Additionally, in spring (March 2022), the isolation rate sharply increased, possibly due to a combination of factors such as warming seasonal temperatures, accelerated reproduction of pathogenic bacteria, and increased breeding density of largemouth bass. Therefore, the monitoring and management of largemouth bass farms in the PRD region should be strengthened during the two critical periods of winter and spring to ensure that potential risks of pathogenic bacterial infections are detected and treated in a timely manner.

Antibiotic resistance has become a global challenge today. Bacterial infections caused by multidrug-resistant or extensively drug-resistant pathogens have emerged as a major issue in clinical treatment worldwide [31,32]. The increase in drug-resistant strains not only results in higher treatment costs but also frequently leads to increased therapeutic side effects and more deaths [33]. This study represents the first analysis of the antibiotic resistance of *E. piscicida* isolated from largemouth bass in the PRD region. The results indicate that the resistance rate to antimicrobials commonly used in aquaculture ranged from 1.25% to 96.25%, with ciprofloxacin exhibiting the highest resistance rate. Notably, 76.25% of these strains were resistant to more than two types of antibiotics. These resistance patterns are similar to those previously observed in fish pathogens within the PRD region [16,34], characterized by high resistance rates and multidrug resistance, further highlighting the severity of antibiotic resistance in aquatic bacteria.

Cluster analysis is commonly used to analyze the correlation of multiple samples with respect to multiple variables and is a statistically significant method for bacterial classification [35], although this method was not previously applied for typing drug-resistant isolates from *E. piscicida*. Further analysis of the 80 *E. piscicida* isolates by clustering the drug sensitivity results classified the isolates into 23 antibiotic types (A–W), 11 subgroups (Ⅰ–XI), and two groups (A and B). These two broad groups showed significant differences in drug resistance. Specifically, the main difference was reported for sulfonamides, with the isolates in Group A exhibiting a higher rate of resistance to sulfonamides than the isolates in Group B, which showed a relatively low rate, and four sulfonamides accounting for 52.17% of the total drug resistance. Sulfonamides are weakly bioconcentrated in fish, but their excessive use poses a great threat to aquatic ecosystems and organisms, leading to the spread of resistant isolates and resistance genes [36]. Oliveira et al. [37] reported that more than 50% of 234 isolates of *Aeromonas* aeruginosa were resistant to sulfonamides. Notably, most of the isolates resistant to two chloramphenicol analogs were also resistant to four sulfonamides, which accounted for 38.75% of the total number of isolates and 26.09% of the total antibiotic spectrum. This finding provides an important basis to understand the differences in resistance among isolates and for the development of targeted treatment programs.

## 5. Conclusions

In this study, the main pathogens in largemouth bass in the PRD region were *E. piscicida*, *A. veronii*, and *N. seriolae*. Notably, *E. piscicida* accounted for the highest proportion of all isolated bacteria, corresponding to 60.92%, and primarily occurred from November to April. *E. piscicida* exhibited varying resistance rates to 11 aquatic antimicrobial drugs, with most of the strains displaying multidrug resistance. The resistance profiles encompassed 23 antibiotic types, which could be divided into two groups. The main differences between the two groups were reflected in their resistance to sulfonamides. This study suggests that aquaculture practitioners should receive education on best practices for antibiotic use and the significance of biosecurity measures in preventing the spread of pathogens. This includes training on proper drug dosage, treatment duration, and the importance of rotating antibiotics to mitigate resistance development. Future research can focus on elucidating the detailed mechanisms of antibiotic resistance encountered in clinical practice, providing specific examples of relevant bacterial pathogens.

## Figures and Tables

**Table 1:** Summary of the samples.

Source of the Samples	Month												Total
	AUG	SEP	OCT	NOV	DEC	JAN	FEB	MAR	APR	MAY	JUN	JUL	
Guangzhou	1	0	2	0	1	2	0	0	0	2	6	1	15
Foshan	40	42	42	25	15	6	7	11	43	49	70	44	394
Zhaoqing	7	5	3	12	0	2	1	3	1	2	3	2	41
Jiangmen	2	3	0	2	0	2	0	5	1	6	1	11	33
Other cities	5	4	3	1	4	4	1	2	2	5	6	12	49
Total	55	54	50	40	20	16	9	21	47	64	86	70	532

Note: Other cities: Shenzhen, Dongguan, Huizhou, Zhuhai, and Zhongshan, i.e., five cities in the Pearl River Delta region that are not major breeding areas for largemouth bass.

**Table 2:** Final concentration (μg/mL) of antibiotics in each well.

	THI	FLO	FLU	ENR	CIP	DOX	NEO	SSM	SME	SDI	SMT
A	64	64	64	8	64	128	64	512	320	384	384
B	32	32	32	4	32	64	32	256	160	192	192
C	16	16	16	2	16	32	16	128	80	96	96
D	8	8	8	1	8	16	8	64	40	48	48
E	4	4	4	0.5	4	8	4	32	20	24	24
F	2	2	2	0.25	2	4	2	16	10	12	12
G	1	1	1	0.125	1	2	1	8	5	6	6
H	0.5	0.5	0.5	0.0625	0.5	1	0.5	4	2.5	3	3

Abbreviations: THI, thiamphenicol; FLO, florfenicol; FLU, flumequine; ENR, enrofloxacin; CIP, ciprofloxacin; DOX, doxycycline; NEO, neomycin; SSM, sodium sulfamonomethoxine; SME, sulfamethoxazole; SDI, sulfadiazine; SMT, sulfamethazine.

**Table 3:** Antibiogram types of the 80 *Edwardsiella piscicida* strains.

Type	No. of Strains	Antibiogram
A	2	THI/CIP/SSM
B	2	THI/FLO/CIP/SSM/SME/SDI/SMT
C	1	THI/FLO/SSM
D	19	CIP
E	13	THI/FLO/ENR/CIP/DOX/SSM/SME/SDI/SMT
F	1	FLU/ENR/CIP/DOX/SSM/SME/SDI/SMT
G	3	THI/CIP
H	7	THI/FLO/ENR/CIP/SSM/SME/SDI/SMT
I	5	THI/CIP/ SSM/SME/SDI/SMT
J	5	CIP/SSM/SME/SDI/SMT
K	1	ENR/CIP/SME/SDI/SMT
L	2	CIP/SME/SDI/SMT
M	1	THI/FLO/FLU/ENR/CIP/DOX/NEO/SSM/SME/SDI/SMT
N	1	FLO/FLU/ENR/CIP/DOX/SSM/SME/SDI/SMT
O	1	THI/FLO/FLU/CIP
P	1	THI/FLO/CIP/DOX/SSM/SME/SDI/SMT
Q	7	THI/FLO/FLU/ENR/CIP/DOX/SSM/SME/SDI/SMT
R	1	THI/FLU/ENR/CIP/DOX/SSM/SME/SDI/SMT
S	1	CIP/SMT
T	1	ENR/CIP/DOX/SSM/SME/SDI/SMT
U	1	CIP/SSM
V	1	THI/CIP/SSM/SMT
W	1	FLU/CIP

Abbreviations: THI, thiamphenicol; FLO, florfenicol; FLU, flumequine; ENR, enrofloxacin; CIP, ciprofloxacin; DOX, doxycycline; NEO, neomycin; SSM, sodium sulfamonomethoxine; SME, sulfamethoxazole; SDI, sulfadiazine; SMT, sulfamethazine.

## Data Availability

The original contributions presented in the study are included in the article, further inquiries can be directed to the corresponding author.
